# Chemical Bonding and Dynamic Structural Fluxionality of a Boron-Based B_8_Al_3_^+^ Cluster

**DOI:** 10.3390/molecules29245961

**Published:** 2024-12-17

**Authors:** Shu-Juan Gao, Tan-Lai Yu

**Affiliations:** 1 Department of Chemical and Materials Engineering, Lyuliang University, Lishi 033001, China; 2 Institute of New Carbon-Based Materials and Zero-Carbon and Negative-Carbon Technology, Lyuliang University, Lishi 033001, China

**Keywords:** dual π/σ aromaticity, dynamic structural fluxionality, chemical bonding, theoretical chemistry

## Abstract

We studied the boron-based composite cluster B_8_Al_3_^+^ doped with Al atoms. The global minimum structure of the B_8_Al_3_^+^ cluster is a three-layer structure, consisting of three parts: an Al_2_ unit, a B_8_ ring and an isolated Al atom. Charge calculations analysis shows that the cluster can be expressed as [Al]^+^[B_8_]^2−^[Al_2_]^2+^, has 6π/6σ double aromaticity and follows the (4*n*+2) Hückel rule. Born–Oppenheimer molecular dynamics (BOMD) simulation shows that the B_8_Al_3_^+^ cluster has dynamic fluxionality properties. Remarkably, at the single-point coupled cluster singles, doubles and triples (CCSD(T)) level, the energy barrier for intramolecular rotation is merely 0.19 kcal mol^−1^. [B_8_]^2−^ molecular wheels have magical 6π/6σ double aromaticity properties, providing a continuous cloud of delocalized electrons, which is a key factor in the dynamic fluxionality of the cluster. The B_8_Al_3_^+^ cluster provides a new example of dynamic structural fluxionality in molecular systems.

## 1. Introduction

Boron, which is positioned nearest to carbon in the periodic table, shares various structural attributes and represents a typical electron-deficient element. Experimental and theoretical investigations of elemental boron clusters have been ongoing since the 1980s [1,2,3,4,5,6,7,8,9,10,11,12,13,14,15]. Systematic experimental and computational analyses have revealed boron clusters’ propensity to exhibit planar or quasi-planar structures across diverse size scales. Owing to their electron deficiency, boron-based clusters possess a unique electron distribution orbital structure, enabling the formation of distinct chemical bonds. Within these clusters, chemical bonding is influenced by π/σ aromaticity, antiaromaticity, and conflicting aromaticity, necessitating electron delocalization to counterbalance boron’s inherent electronic deficiencies [4,5,6,9]. Additionally, the unusual bonding mode also leads to dynamic structural fluxionality of bare boron clusters and related compound systems.

Metal atoms are introduced into boron clusters to create boron-based alloy clusters. The approach proves effective in exploring structural diversity, adjusting electronic properties, and uncovering new chemical bonds within these clusters [16,17,18,19,20]. Utilizing intramolecular charge transfer allows precise electron counting in alloy clusters, facilitating the deliberate design of new cluster structures and deeper investigation into their bonding and dynamic attributes. Structural fluxionality is an extraordinary attribute inherent in boron clusters, manifesting as dynamic flexibility. Boron tends to form multicenter bonds in both its compounds and at nanoscales, and fluxionality is attributed to the multicenter bonds found in boron clusters [21]. The electron deficiency of boron contributes significantly to this distinct dynamic behavior. Researchers have consistently designed and documented a range of pure boron clusters showcasing dynamic fluxionality, including B_11_^−^, B_13_^+^, B_15_^+^, and B_19_^−^ [22,23,24,25]. Subsequent research revealed that blending different metals enables the deliberate design of boron-based cluster nanomachines exhibiting dynamic fluxionality. In 2017, Zhai and colleagues identified nearly isoenergetic three-layer and spiral structures within the Be_6_B_11_^−^ cluster [26]. The former sandwich structure demonstrates two dynamic rotation/twisting modes, resembling structural fluidity akin to the nanoscale Earth–Moon system. Furthermore, researchers have observed magical dynamic fluxionality in a range of binary boron-based nanoclusters, including Na_5_B_7_, V_2_B_7_^−^, and Be_3_B_11_^−^ [27,28,29], among others.

To diminish the dynamic energy barrier associated with fluxionality, researchers started incorporating multiple metal atoms to alter the electron distribution within boron clusters. In compass-like clusters MB_7_X_2_ and MB_8_X_2_ (where X stands for Zn or Cd and M represents Be, Ru, or Os) [30], the X_2_ needle undergoes rotation along the B_8_ wheel. The central B_6_ ring within the boron-based ternary Rb_6_Be_2_B_6_ cluster [31], featuring a distinct sandwich structure, emerges as exposed bare B enclosed by two tetrahedral BeRb_3_ ligands. The bonding pattern within the vast sandwich cluster encourages distinctive dual-mode dynamic fluxionality. The study aims to examine the structure of the ternary cluster B_8_Al_3_^+^, analyze its structural stability, and determine if it presents novel instances of dynamic structural fluxionality. The GM structure of the B_8_Al_3_^+^ was determined using a CK search. The B_8_Al_3_^+^ cluster exhibits a three-layer structure. Its geometric configuration resembles a “clock”: the middle B_8_ ring creates the dial, while the two aluminum atoms above it act as pointers, with an Al unit below them. Chemical bonding analysis indicates that the B_8_Al_3_^+^ cluster exhibits 6π/6σ dual aromaticity, with a double delocalized electron cloud facilitating a continuous “orbit,” enabling unrestricted rotation of the Al_2_ unit above the B_8_ ring. Charge calculations reveal evident charge transfer among the Al_2_ unit, the Al atom and the B_8_ ring, presenting a formal description as a [Al]^+^[B_8_]^2−^[Al_2_]^2+^ ion complex.

## 2. Results

### 2.1. Global Minimum of B_8_Al_3_^+^ Cluster

**Figure 1:**
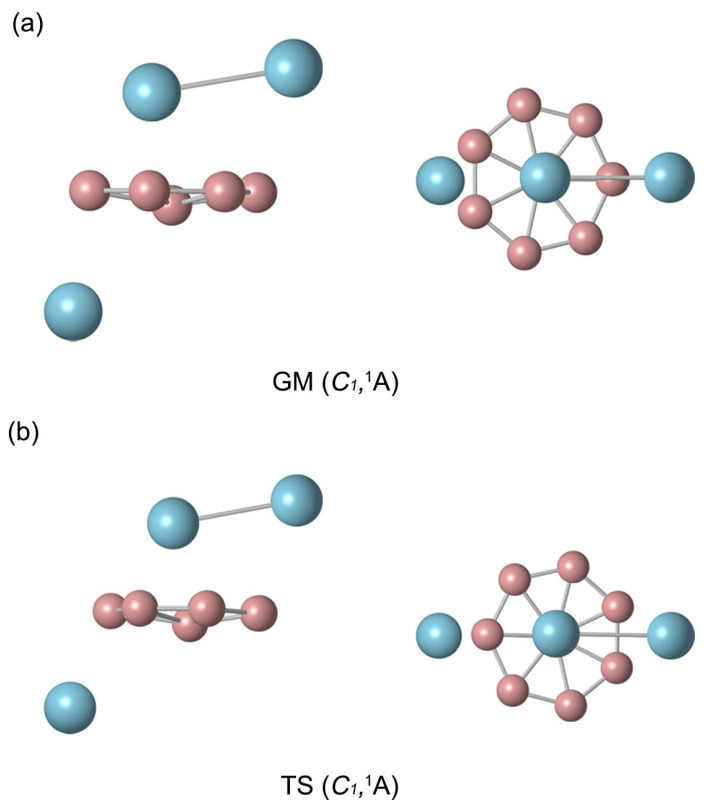
Optimized (**a**) *C*_1_ global minimum (GM) and (**b**) *C*_1_ transition state (TS) structures of B_8_Al_3_^+^ cluster at the PBE0/def2-TZVP level. Presented in top and side views. The B and Al atoms are in pink and blue, respectively. The same below.

Figure 1a shows the GM *C*_1_ (^1^A) structure of the B_8_Al_3_^+^ cluster. The relative energies of the top 20 low-lying isomers, including zero-point energy correction (ZPE), are provided in Figure S1. The B_8_Al_3_^+^ cluster exhibits *C*_1_ symmetry, signifying the global minimum on the potential energy surface. Initial computations using PBE0/def2-TZVP on the top 20 structures revealed the GM cluster to be 1.01 kcal mol^−1^ lower in energy than its nearest competitor. It is worth noting that within the DFT method, the PBE0 and B3LYP functionals are widely acknowledged for their complementarity in molecular systems. To ensure computational consistency across density functionals concerning geometry and energetics, we completed a comparison at the B3LYP/def2-TZVP method level, revealing the GM cluster to be 7.71 kcal mol^−1^ lower in energy than its closest competitor. Subsequent calculations conducted at the CCSD(T)/def2-TZVP//PBE0/def2-TZVP and complementary CCSD(T)/def2-TZVP//B3LYP/def2-TZVP levels indicated energy advantages of 5.61 kcal mol^−1^ and 4.65 kcal mol^−1^, respectively, for the GM structure. Hence, based on the aforementioned data, the GM *C*_1_ (^1^A) structure of B_8_Al_3_^+^ cluster is confirmed to be a really minimal structure on the potential energy surface.

Figure 1a displays the top and side views of the GM B_8_Al_3_^+^ cluster, representing a closed-shell electronic system. The GM B_8_Al_3_^+^ cluster comprises three layers, resembling a clock: the middle B_8_ ring creates the dial, while the two aluminum atoms above it act as pointers, with an Al unit below it. Figure 1b demonstrates the similarity between the TS structure and the GM structure, where the Al–Al pointer rotates approximately 25.7° to achieve the TS structure. Currently, the Al–Al pointer is positioned between the two B atoms on the dial. The cartesian coordinates of GM cluster and TS structure at PBE0/def2-TZVP are presented in Table S1.

### 2.2. Bond Distances, Wiberg Bond Indices, and Natural Atomic Charges

**Figure 2:**
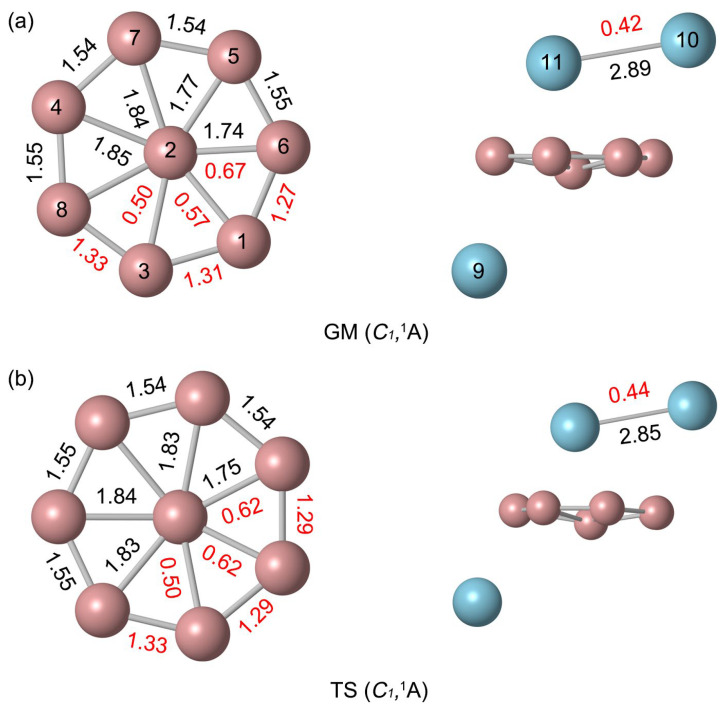
Calculated bond distances (in Å, black color) and Wiberg bond indices (WBIs, in red color) for (**a**) C_1_ (^1^A) GM and (**b**) C_1_ (^1^A) TS structures of B_8_Al_3_^+^ cluster at the PBE0/def2-TZVP level. The WBIs are obtained from the natural bond orbital (NBO) analysis at PBE0/def2-TZVP.

Figure 2a displays the bond distances and bond orders for the GM B_8_Al_3_^+^ cluster. The boron ring’s peripheral bond distances are 1.54–1.55 Å, below the upper bound of 1.70 Å for B–B single bonds. This suggests that there is a force between the B atoms involving both covalent single bonds and delocalized electrons. The bond orders of the B_8_ ring are greater than 1, which also illustrates this essence. Radial B–B links are much longer (1.74–1.85 Å); the links are in line with delocalized π/σ bonding, and are weaker than a single bond. Indeed, their calculated WBIs amount to 0.50–0.67. The distance between the two Al atoms, 2.89 Å, is close to the upper limit of the Al–Al single bond (2.52 Å), and the corresponding Al–Al bond order is 0.42, indicating the presence of an Al–Al single bond.

**Figure 3:**
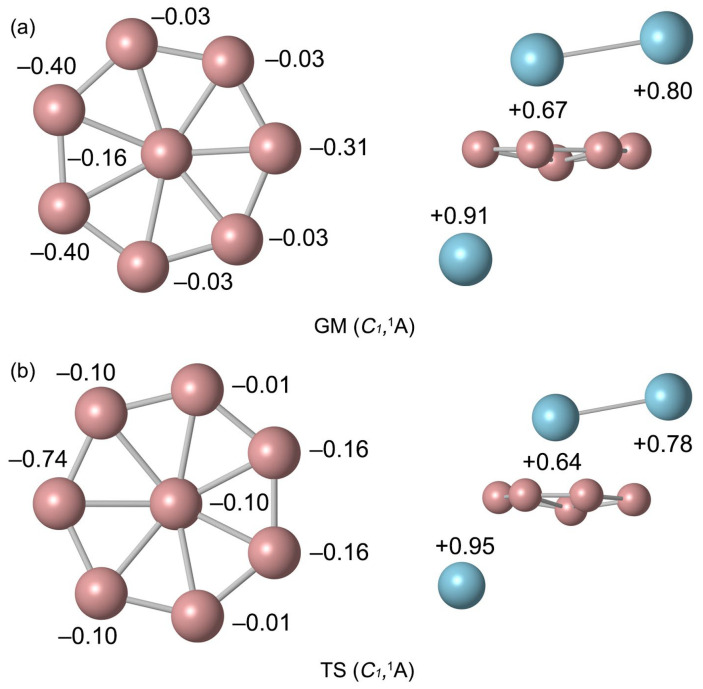
Natural atomic charges (in |e|) for (**a**) *C*_1_ (^1^A) GM and (**b**) *C*_1_ (^1^A) TS structures of B_8_Al_3_^+^ cluster. The data are obtained from the NBO analyses at PBE0/def2-TZVP.

The natural atomic charges were calculated by natural bond orbital (NBO) analysis, as shown in Figure 3. The Al9 atom below the B ring carries a positive charge of +0.91 |e|. These natural atomic charge data indicate that one electron is transferred from the Al atom to the B_8_ ring. The rightmost B6 atom near the pointer Al_2_ carries a charge of −0.31 |e|, indicating an electrostatic interaction between the B and Al atoms (Figure 3a). The B atoms are negatively charged from −0.03 to −0.40 |e| in GM and from −0.01 to −0.74 |e| in TS. The charge carried by Al_2_ unit is +1.47 |e|, which can be regarded as transferring approximately two electrons to the B_8_ ring. The bond distances and bond order of TS and GM are similar. Due to the deflection of the Al needle, the natural atomic charge changes slightly. Overall, the charge transfer case is identical. The similarity in structure and chemical bonding of GM and TS implies that there is a lower energy barrier between them. Thus, both the GM and TS structures of B_8_Al_3_^+^ cluster are essential charge transfer complexes of [Al]^+^[B_8_]^2−^[Al_2_]^2+^.

## 3. Discussion

### 3.1. Dynamic Structural Fluxionality

Vibration frequency analysis was conducted on the GM and TS structures of B_8_Al_3_^+^ cluster at PBE0 level, as illustrated in Figure S2. The GM B_8_Al_3_^+^ cluster exhibits a vibration soft mode at 30.43 cm^−1^, corresponding to the tangential reverse motion involving the Al_2_ unit and the B_8_ ring. The movement along the vibration soft mode vector leads to the formation of the corresponding TS structure. The TS structure’s vibration soft mode is 29.48*i* cm^−1^. Both exhibit similar rotation modes that facilitate the relative rotation of the Al_2_ unit above the B_8_ ring. These soft vibrational modes are perfectly in line with dynamic structural fluxionality of the system as a molecular rotor.

To vividly demonstrate the dynamic fluxionality of the B_8_Al_3_^+^ cluster, we have run a BOMD simulation at a selected set of temperatures of 300 K for a time span of 50 ps. In the BOMD simulation, we take the GM structure as the initial structure and simulate the dynamic evolution process. The coordinates of the whole molecular dynamics process can be obtained at the set temperature, and the analysis of the results can be performed to obtain the corresponding dynamic properties. The system dynamics trajectories were obtained by taking GM structure as the initial coordinate at PBE0 level. The BOMD data are visualized using GaussView 6.0, which vividly shows that the present ternary cluster behaves similarly to a functioning compass at the subnanoscale. The BOMD simulation results vividly illustrate the dynamic fluxionality process within the B_8_Al_3_^+^ cluster: the Al−Al unit rotates above the B_8_ ring, resembling the motion of a clock hands. Throughout the BOMD simulation, the B_8_Al_3_^+^ cluster consistently retained geometric stability without noticeable deformation. A short video is extracted from the simulation and presented in the ESI. The animation approximately lasts for 12 ps.

**Figure 4:**
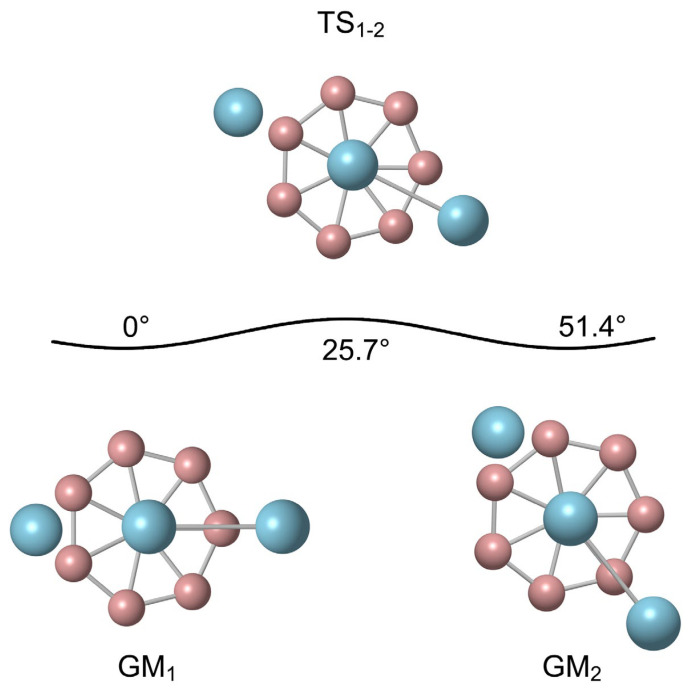
Structural evolution of B_8_Al_3_^+^ cluster during intramolecular dynamic rotation of the Al–Al dimer with respect to B_8_ molecular wheel.

Figure 4 displays the dynamic evolution process of B_8_Al_3_^+^ cluster. The rotation energy barrier for B_8_Al_3_^+^ cluster is 0.32 kcal mol^−1^ at PBE0 level, refined to 0.19 kcal mol^−1^ at the CCSD(T) level. The Al−Al unit, resembling a pointer, is suspended above the B_8_ ring. Starting from GM_1_ as the initial configuration, it rotates clockwise with the B_8_ ring’s center as the axis. As the Al−Al pointer rotates 25.7° clockwise, surpassing the rotation energy barrier, it reaches the first transition state, TS_1-2_, while the Al−Al pointer is perpendicular to the B-B bond of the two adjacent B atoms below at this stage. Upon rotating the Al–Al dimer by another 25.7° clockwise, one recovers the GM geometry (GM_2_). Subsequently, it continues to rotate clockwise, always exceeding the reaction energy barrier. After completing the above process using six TS configurations and five GM configurations, all atoms in the B_8_Al_3_^+^ cluster eventually return to their initial positions.

### 3.2. Chemical Bonding

**Figure 5:**
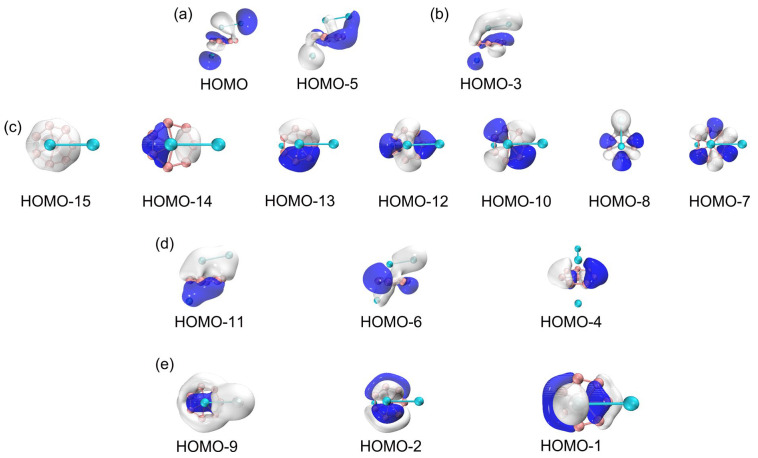
Pictures of occupied canonical molecular orbitals (CMOs) of GM B_8_Al_3_^+^ cluster. (**a**) Lone pairs. (**b**) Lewis-type Al–Al σ bond. (**c**) Seven CMOs for Lewis B–B σ single bonds along the periphery of disk B_8_ motif. (**d**) Three delocalized π CMOs. (**e**) Three delocalized σ CMOs.

To understand the unique geometries, stability, and dynamic fluxionality of GM B_8_Al_3_^+^ cluster, it is essential to elucidate their chemical bonding. For this purpose, CMOs and AdNDP analyses are fundamental. The GM B_8_Al_3_^+^ cluster is a closed-shell cluster with 32 valence electrons. Its 16 occupied CMOs are sorted to five subsets based on their constituent atomic orbitals (AOs), as shown in Figure 5. The two CMOs in subset (a) are composed mainly of 3s/3p AOs from two aluminum atoms, in their constructive versus destructive combinations. According to the CMO construction principles, these two CMOs can be localized as the lone pairs of Al 3s^2^. HOMO–3 in subset (b) is responsible for an interlayer Al–Al σ single bond, which originates from 3s AOs of two Al atoms. Meanwhile, the 14 orbital electrons in subset (c) are derived mainly from the 2s and 2p atomic orbitals of the B_8_ ring.

**Figure 6:**
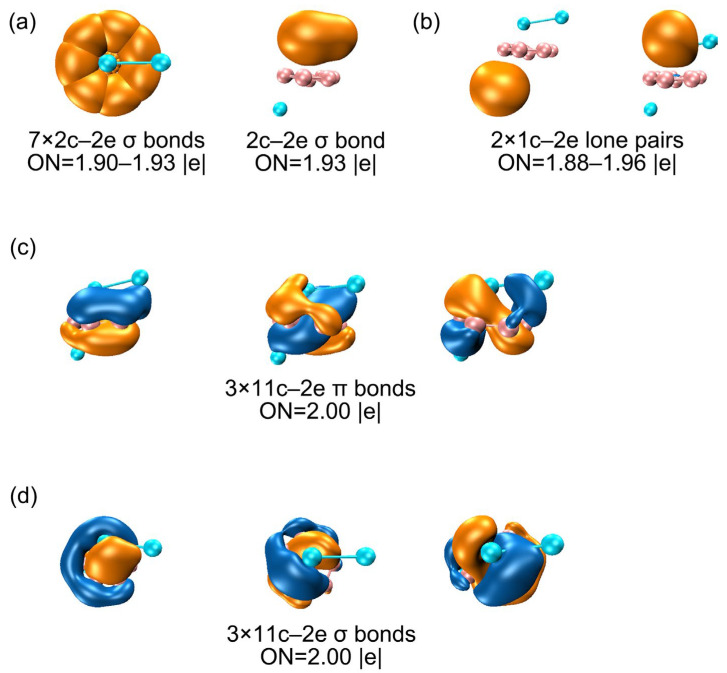
AdNDP bonding scheme for GM (*C*_1_, ^1^A) B_8_Al_3_^+^ cluster. (**a**) Seven quasi-Lewis-type 2c-2e B–B σ bonds along the periphery and a Al–Al σ bond. (**b**) Two lone pairs of Al. (**c**) Global π sextet. (**d**) Global σ sextet. Occupation numbers (ONs) are shown.

There are seven CMOs in subset (c) containing HOMO–15, HOMO–14, HOMO–13, HOMO–12, HOMO–10, HOMO–8 and HOMO–7, which constitute a complete series with 0–3 nodal planes (sequentially from left to right), including 2 degenerate pairs. Upon recombination, these seven orbitals can form seven two-center two-electron (2c–2e) bonds, thereby being localized as seven B–B σ bonds. These Lewis-type bonds constitute the cluster’s structural framework, utilizing a total of 14 electrons. Figure 6a shows the AdNDP bonding scheme, affirming the alignment between seven CMOs and the seven 2c–2e single bonds on the B_8_ ring. The occupation numbers (ONs) are 1.90–1.93 |e|, which are generally close to the ideal value of 2.00 |e|. Subset (d) is the cluster’s delocalized π framework, which is primarily sourced from the 2s/2p orbitals of B atoms (Table S2). The corresponding AdNDP bonding scheme allocates three delocalized π orbitals into three 11c–2e bonds, with an occupation value of 2.00 |e|. Three CMOs in subset (e) constitute a delocalized 6σ subsystem, situated on the eight B atoms. Subsets (d) and (e) each comprise three orbitals and six delocalized electrons, satisfying the (4*n*+2) Hückel rule (*n* = 1), establishing the cluster’s π/σ dual aromaticity. In subset (b), an Al–Al σ orbital aligns with AdNDP bonding principles, corresponding to the AdNDP scheme in Figure 6a (right). In the TS structure, the CMOs, AdNDP scheme and orbital compositions remain virtually unaltered (Figures S3 and S4, ESI†), which explains why the dynamic fluxionality process has no energy barrier.

In summary, the chemical bonding of the B_8_Al_3_^+^ cluster consists of the lone pairs of two Al atoms, a covalent Al–Al single bond, seven 2c–2e Lewis single bonds within the B8 ring, three 11-center delocalized σ and three 11-center delocalized π bonds, which establishes their two-fold 6π/6σ aromaticity. This dual aromaticity collectively underlies the unique dynamic fluxionality of the cluster.

**Figure 7:**
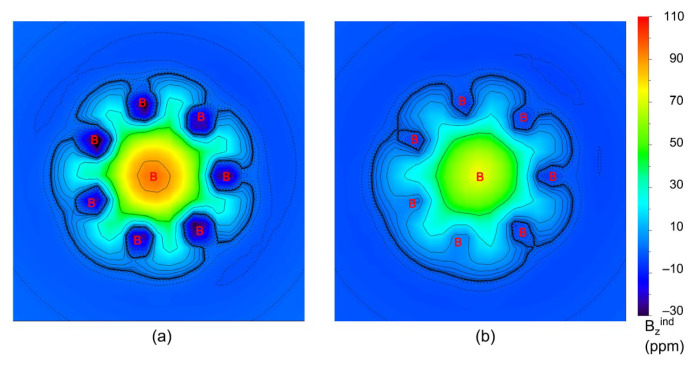
The iso-chemical shielding surfaces (ICSSs) of GM B_8_Al_3_^+^ cluster. (**a**) ICSS_zz_(0) at the B_8_ disk plane. (**b**) ICSS_zz_(1) at 1.0 Å below the disk plane. For ICSSs, a positive value indicates aromaticity, and vice versa.

To more intuitively observe the dual 6π/6σ aromaticity, the color-filled maps of ICSS_zz_ of B_8_Al_3_^+^ cluster at 0 (ICSS_zz_(0)) and 1 Å (ICSS_zz_(1)) above the B_8_ ring plane are plotted in Figure 7. The green areas in (a) and (b) within the molecular wheel, in which the shielding effect is primarily concentrated, are in line with σ and π aromaticity of the cluster, respectively. Obviously, the [B_8_]^2−^ unit is doubly 6π/6σ aromatic, which is the key factor that causes the ground state to stabilize. In essence, 6π/6σ double aromaticity of [B_8_]^2−^ is based on its charge transfer, as described previously. Nucleus-independent chemical shifts, NICS and NICS_zz_, are calculated for the GM B_8_Al_3_^+^ cluster as additional criteria for aromaticity (Table S3, ESI). The large negative values are consistent with the assessment of π and σ double aromaticity. The NICS and NICSzz values at the center of a B_3_ triangle are actually helpful for understanding the aromaticity of GM B_8_Al_3_^+^, whereas those at 1 Å below the plane probe π aromaticity. The electron clouds over the system are uniform and dilute facilitate dynamic fluxionality; these effects are based on two-fold magic 6π/6σ aromaticity.

## 4. Methods

The global minimum (GM) structure and low-lying isomers of the B_8_Al_3_^+^ cluster were determined through a Coalescence Kick (CK) search and more than 5000 stationary points (3000 singlet and 2000 triplet) were detected on the potential energy surface with the help of artificial structure construction [32,33]. The candidate low-lying structures were subsequently reoptimized at the PBE0/def2-TZVP level [34,35]. Frequency calculations were carried out at the same level to ensure that the reported structures are true minima. To check for the computational consistency of different functionals in structures and energetics, the zero-point correction energy was also calculated at the B3LYP/PBE0/def2-TZVP. In order to benchmark the relative energies, the top five low-lying isomers were further assessed at the single-point CCSD(T)/def2-TZVP level on the basis of their PBE0/def2-TZVP and B3LYP/def2-TZVP geometries [36,37,38].

At the PBE0/def2-TZVP level, orbital composition analysis was completed through NAO calculations, and Wiberg bond indices (WBIs) and natural atomic charges were obtained through natural bond orbital (NBO) calculations [39]. Chemical bonds were elucidated using canonical molecular orbital (CMO) analysis and adaptive natural density partitioning (AdNDP) [40]. Nucleus-independent chemical shifts (NICSs) and iso-chemical shielding surfaces (ICSSs) were calculated to evaluate π/σ aromaticity [41,42]. AdNDP analysis and ICSSs calculations were performed using the Multifwn program [43]. We performed Born–Oppenheimer molecular dynamics (BOMD) simulations at a temperature of 300 K to study the dynamic properties of the clusters [44]. All the above calculations were performed using the Gaussian 09 software package [45]. The visualization of the calculation results was completed through GaussView 6.0, CYLview 1.0 and VMD 1.9.4 programs [46,47,48].

## 5. Conclusions

In summary, we have designed a cationic ternary boron-based binary B–Al cluster, B_8_Al_3_^+^, which adopted a three-layered structure in a subnanoscale clock shape with a quasi-planar B_8_ wheel. The Al_2_ pointer is above the wheel and there is another aluminum atom under it. It features dynamic structural fluxionality at 300 K. Charge calculations suggest that the cluster can be described as a charge transfer ion complex and formulated as [Al]^+^[B_8_]^2−^[Al_2_]^2+^, whose three charged layers are bound via quite strong electrostatic forces. BOMD simulation indicates that the Al–Al pointer can freely rotate on the dial below, requiring an energy barrier of 0.19 kcal mol^−1^ to facilitate its rotation. Chemical bonding analyses indicate the magic 6π/6σ double aromaticity of the B_8_Al_3_^+^ cluster. The dual delocalized electron clouds create a fluid “orbital” that allows the Al_2_ unit above the B_8_ ring to rotate freely, thus imparting dynamism to the cluster. The balance of electrostatic traction and repulsion between layers is critical to the dynamic fluxionality of the cluster. The stability of molecular wheels and molecular motors is contingent upon the cluster structure, size, bonding mode, and charge state. A deeper understanding of how delocalized electrons change and flow during the rotation of a molecular motor, and of the factors determining the level of rotational energy barriers, would be beneficial. Similarly, further insight into the interaction mechanism between a molecular motor and an electron beam, and its effect on rotational energy barriers, would be valuable. Subsequently, doping strategies or electron compensation strategies may be employed to transform rigid boron radicals into structurally rheological boron radicals. It will be possible to initiate or terminate the operation of molecular motors by adjusting the temperature, electric field, magnetic field, and so forth. Based on this, it will be possible to further develop valuable functionalized molecular machines. Examples include nano cars, nano trucks, one-wheeled nano cars and walking robots.

## Data Availability

Data are contained within the article and Supplementary Materials.

## Supplementary Materials

The following supporting information can be downloaded at https://www.mdpi.com/article/10.3390/molecules29245961/s1. Table S1: Cartesian coordinates for optimized global minimum (GM) and transition state (TS) structures of the B_8_Al_3_^+^ cluster at the PBE0/def2-TZVP level; Table S2: Orbital composition analyses of occupied canonical molecular orbitals (CMOs) of GM (*C*_1_, ^1^A) B_8_Al_3_^+^ cluster; Table S3: Calculated NICS_zz_ and NICS (shown in italics in brackets) of GM B_8_Al_3_^+^ cluster at the PBE0/def2-TZVP level. These values are calculated at the center of the B_3_ triangle, as well as at 1 Å above the center; Table S4: Vibrational frequencies of the B_8_Al_3_^+^ cluster at the PBE0/def2-TZVP level; Figure S1: Alternative optimized structures for the B_8_Al_3_^+^ cluster at the PBE0/def2-TZVP level including zero-point energy (ZPE) corrections, along with their relative energies. Relative energies are also presented for top five lowest-energy isomers at the single-point CCSD(T)/def2-TZVP//PBE0/def2-TZVP (in parentheses) and for the top two lowest-energy isomers at the B3LYP/def2-TZVP (in square brackets, with ZPE corrections), and single-point CCSD(T)/def2-TZVP//B3LYP/def2-TZVP (in curly brackets) levels of theory. All energies are shown in kcal mol^−1^; Figure S2: Displacement vectors of the vibrational modes of (a) GM and (b) TS structures of the B_8_Al_3_^+^ cluster at the PBE0/def2-TZVP level; Figure S3: Pictures of occupied canonical molecular orbitals (CMOs) of TS B_8_Al_3_^+^ cluster. (a) Lone pairs. (b) Lewis-type Al–Al σ bond. (c) Seven CMOs for Lewis B–B σ single bonds along the periphery of disk B8 motif. (d) Three delocalized π CMOs. (e) Three delocalized σ CMOs; Figure S4: AdNDP bonding scheme for TS (*C*_1_, ^1^A) B_8_Al_3_^+^ cluster. Occupation numbers (ONs) are shown.
