# Supplementary material for: Chemical Bonding and Dynamic Structural Fluxionality of a Boron-Based B8Al3+ Cluster
Source: Molecules. 2024 Dec 17;29(24):5961. doi: 10.3390/molecules29245961 (PMC11679524; doi:10.3390/molecules29245961)

SUPPLEMENTARY INFORMATION

Chemical Bonding and Dynamic Structural Fluxionality of a Boron-Based $B_8Al_3^+$ Cluster

Shu-Juan Gao [1,2,*] and Tan-Lai Yu [1,2,*]

[1]Department of Chemical and Materials Engineering, Lyuliang University, Lishi Shanxi 033001, China

[2]Institute of New Carbon-based Materials and Zero-carbon and Negative-carbon Technology, Lyuliang University, Lishi Shanxi 033001, China

* Correspondence: shujuangao@llu.edu.cn (S.-J.G.); 20171017@llu.edu.cn (T.-L.Y.).

Supplementary Information – Part I

Table S1. Cartesian coordinates for optimized global-minimum (GM) and transition-state (TS) structures of $B_8Al_3^+$ cluster at the PBE0/def2-TZVP level.

Table S2. Orbital composition analyses for occupied canonical molecular orbitals (CMOs) of GM (C_1, ^{1}A) $B_8Al_3^+$ cluster.

Table S3. Calculated $NICS_{zz}$ and NICS (shown in italics in brackets) of GM $B_8Al_3^+$ cluster at the PBE0/def2-TZVP level. These values are calculated at the center of B_3 triangle, as well as at 1 Å above the center.

Table S4. Table of vibrational frequencies of $B_8Al_3^+$ cluster at the PBE0/def2-TZVP level.

Figure S1. Alternative optimized structures for $B_8Al_3^+$ cluster at the PBE0/def2-TZVP level including zero-point energy (ZPE) corrections, along with their relative energies. The back italics represent the energy after the D3 correction. Relative energies are also presented for top 5 lowest-energy isomers at the single-point CCSD(T)/def2-TZVP//PBE0/def2-TZVP (in parentheses) and for top 5 lowest-energy isomers at the B3LYP/def2-TZVP (in square brackets, with ZPE corrections), and single-point CCSD(T)/def2-TZVP//B3LYP/def2-TZVP (in curly brackets) levels of theory. All energies are shown in kcal mol^{-1}.

Figure S2. Displacement vectors of the vibrational modes of (a) GM and (b) TS structures of the $B_8Al_3^+$ cluster at the PBE0/def2-TZVP level.

Figure S3. Pictures of occupied canonical molecular orbitals (CMOs) of TS $B_8Al_3^+$ cluster. (a) Lone pairs. (b) Lewis-type Al–Al σ bond. (c) Seven CMOs for Lewis B–B σ single bonds along the periphery of disk B8 motif. (d) Three delocalized π CMOs. (e) Three delocalized σ CMOs.

Figure S4. AdNDP bonding scheme for TS (C_1, ^{1}A) $B_8Al_3^+$ cluster. Occupation numbers (ONs) are shown.

Supplementary Information – Part II

A short movie extracted from the BOMD simulation for $B_8Al_3^+$ cluster. The simulation has been performed at near room temperature (300 K) for 50 ps. The movie roughly covers a time span of 10 ps.

Table S1. Cartesian coordinates for optimized global-minimum (GM) and transition-state (TS) structures of $B_8Al_3^+$ cluster at the PBE0/def2-TZVP level.

GM, $B_8Al_3^+$ (C_1, ^1A)

B	-0.49003800	-0.99437000	-1.39845200
B	0.42715000	-0.42655500	0.00038600
B	0.52912100	0.10353400	-1.75359300
B	1.34534600	0.97435400	0.77453800
B	-0.49038400	-0.99309800	1.39947600
B	-0.94072600	-1.49121300	0.00068000
B	0.52870800	0.10510600	1.75390100
B	1.34552900	0.97366500	-0.77481000
Al	3.34285100	-0.45776400	-0.00000100
Al	-3.26435200	-0.30382300	-0.00008900
Al	-0.94569300	1.43411700	-0.00072800

TS, $B_8Al_3^+$ (C_1, ^1A)

B	1.06098000	-1.47137100	0.75498400
B	-0.28935900	-0.66585400	-0.00789000
B	0.18906700	-0.68432900	1.74990700
B	-1.51173500	0.66217700	0.00887900
B	0.18776500	-0.64107200	-1.76618400
B	1.06049200	-1.45230300	-0.79158800
B	-0.95280800	0.31559200	-1.39350200
B	-0.95171700	0.28129100	1.40182900

Al	-3.77588000	-0.10031400	-0.00091300
Al	3.41385900	0.07526300	0.00146400
Al	0.82637300	1.43115500	0.01620400

Table S2. Orbital composition analysis for occupied canonical molecular orbitals (CMOs) of GM (C_1, ^{1}A) $B_8Al_3^+$ cluster.

Subsystem	CMO	B_8(%)		Al_9(%)		Al_{10}(%)		Al_{11}(%)	
		s/p	total	s/p	total	s/p	total	s/p	total
B-B 2c-2e σ	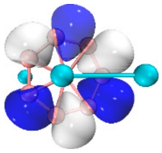 HOMO-7	6.9/91.1	98.0	0.0/0.0	0.0	0.0/0.0	0.0	0.0/0.0	0.0
	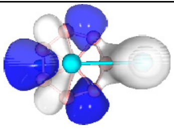 HOMO-8	8.1/85.0	93.1	0.8/0.0	0.8	2.9/0.0	2.9	0.0/0.0	0.0
	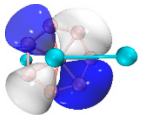 HOMO-10	34.0/62.8	96.8	0.0/0.0	0.0	0.0/0.0	0.0	0.0/0.0	0.0
	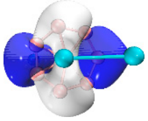 HOMO-12	29.6/62.6	92.2	3.3/0.0	3.3	0.8/0.0	0.8	0.8/0.0	0.8
	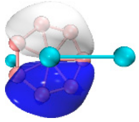 HOMO-13	37.4/58.7	96.1	0.0/0.0	0.0	0.0/0.0	0.0	0.0/0.0	0.0
	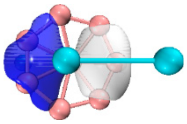 HOMO-14	37.7/56.2	93.9	0.0/0.0	0.0	0.0/0.0	0.0	0.0/0.5	0.5
	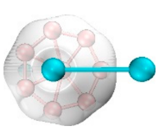 HOMO-15	60.4/35.5	95.9	0.0/0.0	0.0	0.0/0.0	0.0	0.8/1.0	1.8
Al- Al 2c-2e σ	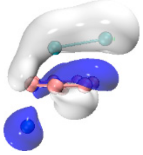 HOMO-3	5.0/40.9	45.9	5.0/0.0	5.0	24.6/2.3	26.9	13.5/3.7	17.2

lone pairs	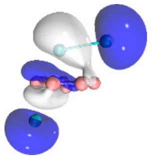 HOMO	0.0/16.4	16.4	13.9/2.2	16.1	18.8/10.7	29.5	27.7/6.0	33.7
	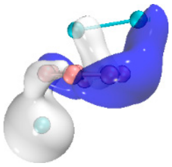 HOMO-5	8.7/36.1	44.8	41.3/0.0	41.3	3.3/0.7	4.0	7.1/0.0	7.1
6π aromaticity	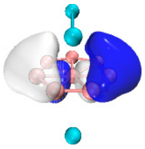 HOMO-4	15.5/76.7	92.2	0.0/0.0	0.0	0.0/0.7	0.7	0.0/5.5	5.5
	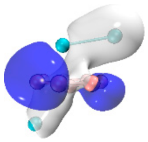 HOMO-6	16.7/44.7	61.4	1.5/0.0	1.5	15.9/0.0	15.9	0.0/5.8	5.8
	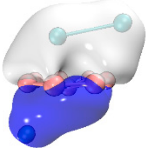 HOMO-11	3.2/67.6	70.8	2.8/0.0	2.8	5.5/0.0	5.5	14.8/0.8	15.6
6σ aromaticity	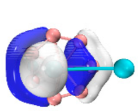 HOMO-1	7.0/48.0	55.0	18.5/0.6	19.1	1.6/0.0	1.6	15.3/1.6	16.9
	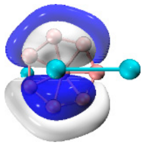 HOMO-2	5.9/85.9	91.8	0.0/1.5	1.5	0.0/0.0	0	0.0/3.3	3.3
	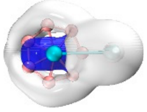 HOMO-9	27.2/40.0	67.2	7.8/0.0	7.8	17.7/0.0	17.7	1.6/1.9	3.5

Table S3. Calculated $NICS_{zz}$ and NICS (shown in italics in brackets) of GM $B_8Al_3^+$ cluster at the PBE0/def2-TZVP level. These values are calculated at the center of B_3 triangle, as well as at 1 Å above the center.

R (Å)	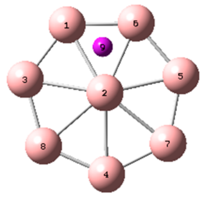	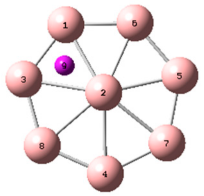	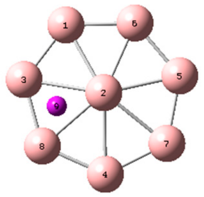	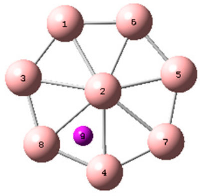
0.0	–38.90<br>(*–57.48*)	–37.13<br>(*–58.57*)	–49.31<br>(*–63.94*)	–45.28<br>(*–60.85*)
1.0	–20.65<br>(*–32.31*)	–22.00<br>(*–31.55*)	–42.84<br>(*–40.15*)	–29.57<br>(*–34.09*)

Table S4. Vibrational frequencies of the $B_8Al_3^+$ cluster at the PBE0/def2-TZVP level.

Mode	Frequency	Infrared	Mode	Frequency	Infrared
27	1419.76	2.73	13	408.11	1.08
26	1374.14	3.71	12	387.05	27.95
25	1251.32	0.40	11	332.71	91.94
24	1246.43	4.51	10	305.23	0.19
23	983.37	21.79	9	254.13	12.65
22	970.19	10.68	8	199.19	16.97
21	819.18	9.86	7	179.98	124.72
20	688.58	8.85	6	128.66	6.57
19	638.84	19.53	5	116.28	2.83
18	585.25	14.77	4	82.35	13.96
17	563.61	15.51	3	59.01	2.29
16	526.48	55.61	2	35.54	5.37
15	470.12	8.19	1	30.43	3.73
14	463.06	1.04			

Figure S1. Alternative optimized structures for $B_8Al_3^+$ cluster at the PBE0/def2-TZVP level including zero-point energy (ZPE) corrections, along with their relative energies. The back italics represent the energy after the D3 correction. Relative energies are also presented for top 5 lowest-energy isomers at the single-point CCSD(T)/def2-TZVP//PBE0/def2-TZVP (in parentheses) and for top 5 lowest-energy isomers at the B3LYP/def2-TZVP (in square brackets, with ZPE corrections), and single-point CCSD(T)/def2-TZVP//B3LYP/def2-TZVP (in curly brackets) levels of theory. All energies are shown in kcal mol^{-1}.

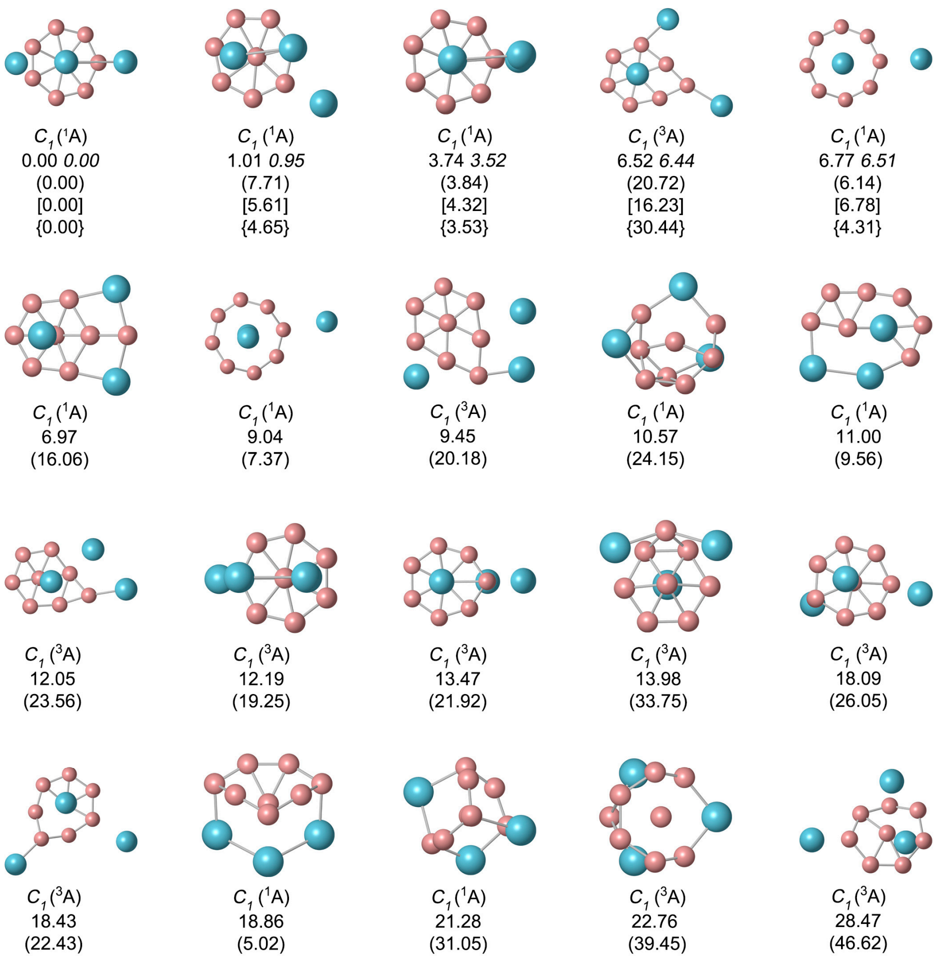

Figure S2. Displacement vectors of the vibrational modes of (a) GM and (b) TS structures of the $B_8Al_3^+$ cluster at the PBE0/def2-TZVP level.

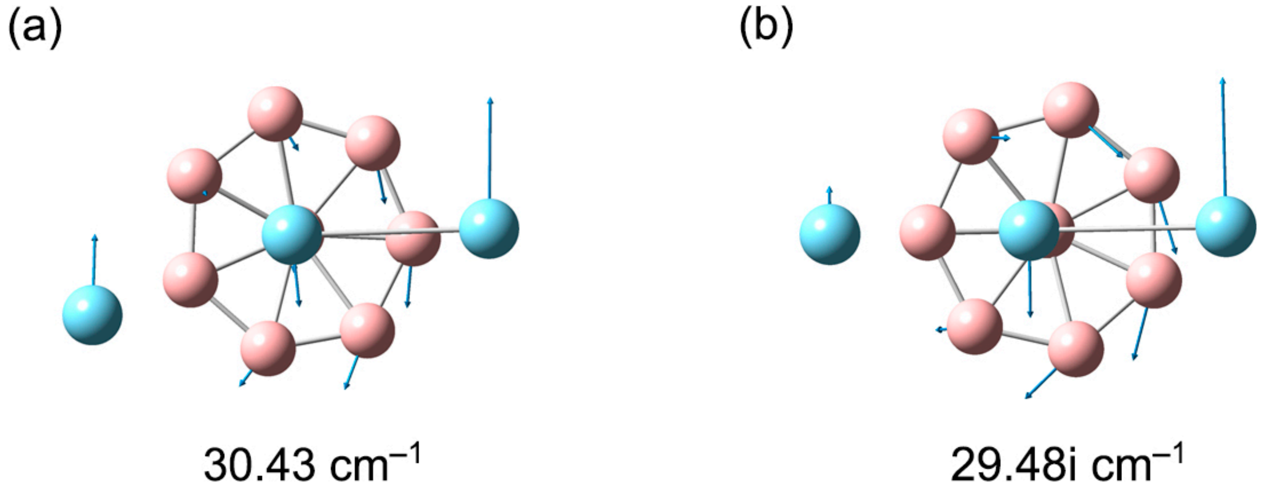

Figure S3. Pictures of occupied canonical molecular orbitals (CMOs) of TS $B_8Al_3^+$ cluster. (a) Lone pairs. (b) Lewis-type Al–Al σ bond. (c) Seven CMOs for lewis B–B σ single bonds along the periphery of disk B8 motif. (d) Three delocalized π CMOs. (e) Three delocalized σ CMOs.

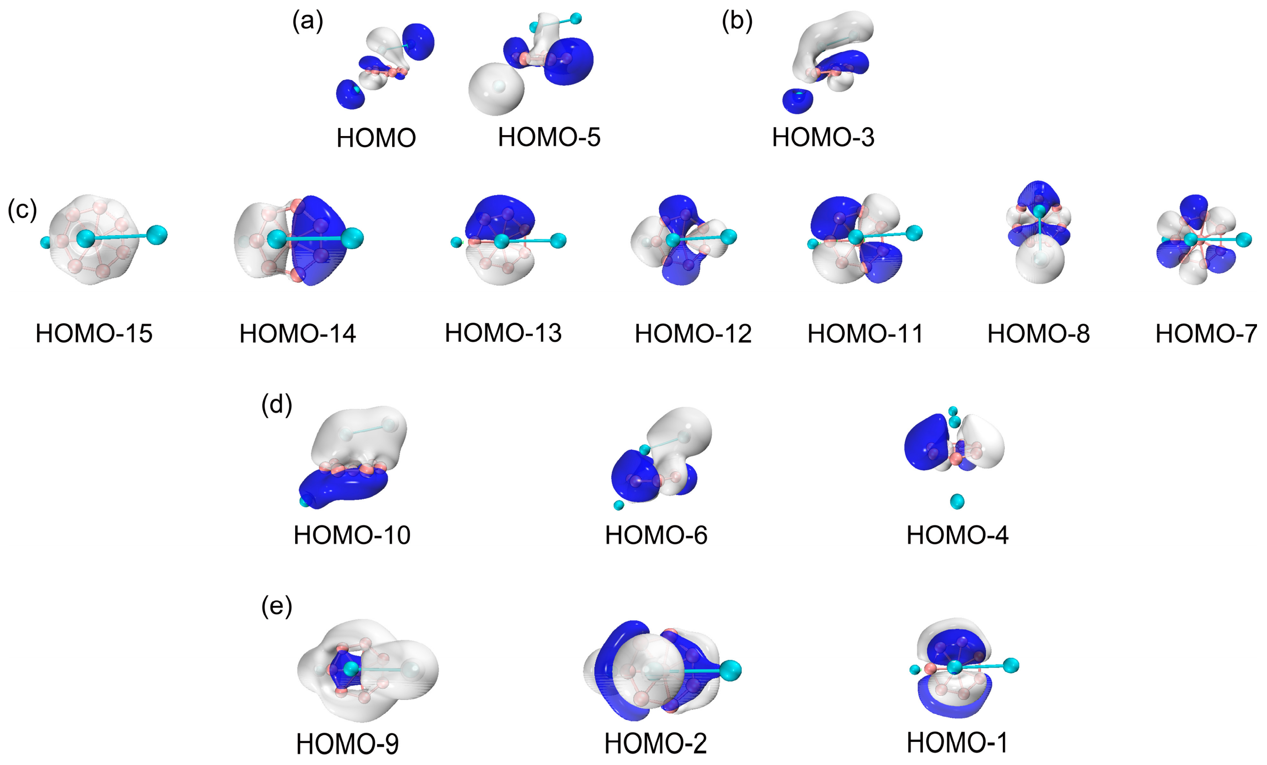

Figure S4. AdNDP bonding scheme for TS (C_1, ^1A) $B_8Al_3^+$ cluster. Occupation numbers (ONs) are shown.

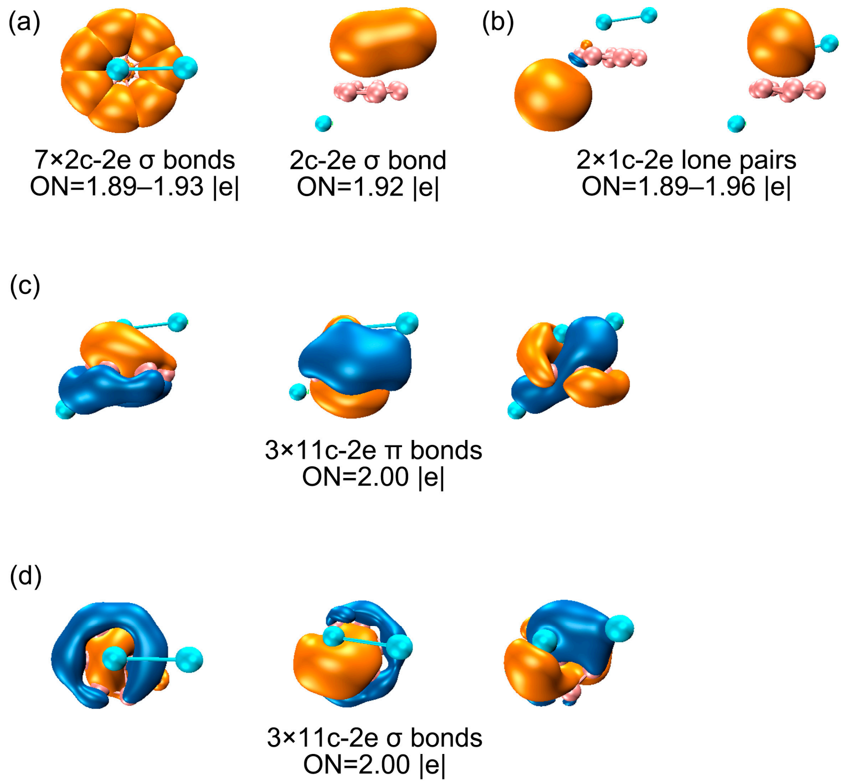

Supplement: Supplementary file 1 [file molecules-29-05961-s001.zip › molecules-3365834-supplementary.pdf]
